# A historic “open book fracture”

**DOI:** 10.11604/pamj.2016.24.9.9352

**Published:** 2016-05-04

**Authors:** Hassen Ben Ghezala, Najla Feriani

**Affiliations:** 1 Service Universitaire des Urgences et de Réanimation Médicale, Hôpital Régional de Zaghouan, Faculté de médecine de Tunis, Tunisie; 2 Service Universitaire de Chirurgie Générale, Hôpital Régional de Zaghouan, Faculté de médecine de Tunis, Tunisie

**Keywords:** Fracture, pelvic ring, surgery

## Image in medicine

“Open book fracture” is rare. It is one of the most dangerous pelvic fractures. It is usually associated with abdominal, vascular and nervous injuries requiring a multidisciplinary team for its management. Its treatment is mainly surgical. Only some cases are published in recent literature. The mechanism is usually complex and the consequences usually dramatic. We report in this case a very rare image of an open book fracture which occurred in a young thirty old man. He was admitted to the emergency department of our hospital after a work accident. He was hit by a heavy (500 kg) charge in his back with impact to the groin in the manufactory where he works. He was transferred by a non-medical transfer to our emergency department. At initial examination, he had a deformed pelvis with moderate bleeding. We observed a large hematoma in his back. The standard pelvis radio X ray (A) revealed a disruption of the pelvic ring with a “third fragment”. Pelvic CT scan showed this third fragment (B). There was a complete fracture of the right iliac wing with a sacroiliac joint disruption. The right and the left halves of the pelvis are separated at front and rear. The front was opening more than the rear. It seemed like an open book and it is called “open book” pelvic fracture. The patient was managed initially in the emergency room with fluid challenge and analgesia. He had then urgent multidisciplinary surgery with external fixation with a good outcome.

**Figure 1:**
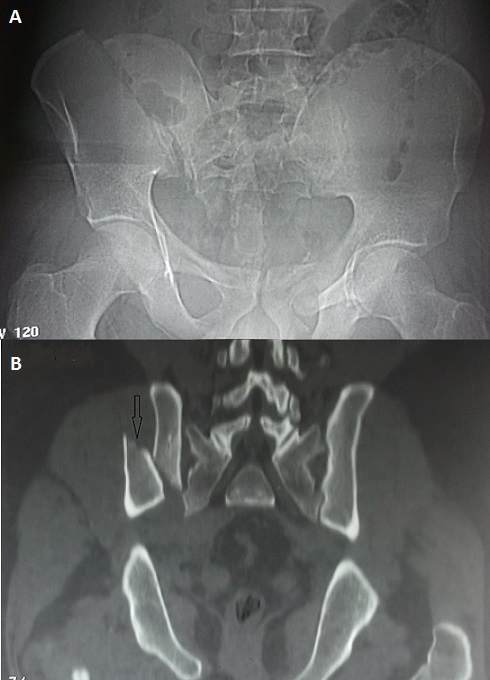
A) pelvis radio X ray showing a complete fracture of the right iliac wing; B) Pelvic CT scan showing the third fragment from the pelvic ring

